# Benefits of ultra-fast-track anesthesia in left ventricular assist device implantation: a retrospective, propensity score matched cohort study of a four-year single center experience

**DOI:** 10.1186/s13019-017-0573-9

**Published:** 2017-02-08

**Authors:** Rashad Zayat, Ares K. Menon, Andreas Goetzenich, Gereon Schaelte, Ruediger Autschbach, Christian Stoppe, Tim-Philipp Simon, Lachmandath Tewarie, Ajay Moza

**Affiliations:** 1 Department of Thoracic and Cardiovascular Surgery, University Hospital RWTH Aachen, Pauwelsstrasse 30, Aachen, 52074 Germany; 2 Department of Anesthesiology, University Hospital RWTH Aachen, Pauwelsstrasse 30, Aachen, 52074 Germany; 3 Department of Intensive Care and Intermediate Care, University Hospital RWTH Aachen, Pauwelsstrasse 30, Aachen, 52074 Germany

**Keywords:** Fast-track-anesthesia, Left ventricular assist device, Right ventricular failure, Postoperative complication

## Abstract

**Background:**

The use of left ventricular assist devices (LVADs) has gained significant importance for treatment of end-stage heart failure. Fast-track procedures are well established in cardiac surgery, whereas knowledge of their benefits after LVAD implantation is sparse. We hypothesized that ultra-fast-track anesthesia (UFTA) with in-theater extubation or at a maximum of 4 h. after surgery is feasible in Interagency Registry for Mechanically Assisted Circulatory Support (INTERMACS) level 3 and 4 patients and might prevent postoperative complications.

**Methods:**

From March, 2010 to March, 2012, 53 LVADs (50 Heart Mate II and 3 Heart Ware) were implanted in patients in our department. UFTA was successfully performed (LVAD_*ultra*_) in 13 patients. After propensity score matching, we compared the LVAD_*ultra*_ group with a matched group (LVAD_*match*_) receiving conventional anesthesia management.

**Results:**

Patients in the LVAD_*ultra*_ group had significantly lower incidences of pneumonia (*p* = 0.031), delirium (*p* = 0.031) and right ventricular failure (RVF) (*p* = 0.031). They showed a significantly higher cardiac index in the first 12 h. (*p* = 0.017); a significantly lower central venous pressure during the first 24 h. postoperatively (*p* = 0.005) and a significantly shorter intensive care unit (ICU) stay (*p* = 0.016). Kaplan-Meier analysis after four years of follow-up showed no significant difference in survival.

**Conclusion:**

In this pilot study, we demonstrated the feasibility of ultra-fast-track anesthesia in LVAD implantation in selected patients with INTERMACS level 3–4. Patients had a lower incidence of postoperative complications, better hemodynamic performance, shorter length of ICU stay and lower incidence of RVF after UFTA. Prospective randomized investigations should examine the preservation of right ventricular function in larger numbers and identify appropriate selection criteria.

**Electronic supplementary material:**

The online version of this article (doi:10.1186/s13019-017-0573-9) contains supplementary material, which is available to authorized users.

## Background

Fast-track anesthesia (FTA) in cardiac surgery had been around long before the nineties but did first gain popularity and acceptance after the 1990s. Many studies showed that, in selected patients, FTA is feasible and safe and reduces the occurrence of ventilator-induced complications, thereby decreasing intensive care unit (ICU) stay, resource use and cost [1–4]. The feasibility of ultra-fast-track anesthesia with in-theater extubation (UFTA) has even been described following heart transplantation and in high-risk patients [5–8]. Prolonged mechanical ventilation is associated with poor outcomes and mortality [9, 10], and it has a deleterious hemodynamic effect first and foremost on right heart function [11–13]. Patients with advanced heart failure requiring left ventricular assist device (LVAD) implantation are particularly prone to many postoperative complications such as respiratory failure, prolonged mechanical ventilation, psychiatric events and right ventricular failure (RVF) leading to high morbidity and mortality [14–16]. Despite ample knowledge of the risk factors promoting right heart dysfunction, RVF remains a serious and dreaded postoperative complication with high mortality rates [14, 15, 17, 18]. In this retrospective study, we aimed to investigate the impact of UFTA following LVAD implantation on ICU and overall hospital stay, and to assess the effect of UFTA in reducing postoperative complication.

## Methods

### Design and data collection

A retrospective data search and analysis of prospectively collected data from all patients who underwent implantation of LVAD between March, 2010 and March, 2012 was performed. Informed consent was waived by our ethical board (Ethik-Komission RWTH) due to the retrospective nature of the analysis. The following data were collected from the electronic database: demographics, comorbidities, preoperative diagnostic results from left and right heart catheterization, echocardiographic findings, spirometry, radiographic finding, laboratory results, perioperative surgical and anesthesia protocols, hemodynamic and ventilation parameters from monitoring during the operation and in the ICU, and packed red blood cells (PRBCs) given in the operating room (OR) and during the remaining hospital stay. European System for Cardiac Operative Risk Evaluation II (EuroSCORE II) and Interagency Registry for Mechanically Assisted Circulatory Support (INTERMACS) were calculated for all patients. Ambulatory patients were routinely followed up every two months from March, 2010 until March, 2016 according to our standardized follow-up protocol for LVAD patients.

### Surgical procedures

All patients underwent cardiac surgery through full median sternotomy. LVAD implantation and, if necessary, concomitant tricuspid valve repair (TVR) and/or coronary artery bypass grafting (CABG) were performed with on-pump beating heart in 38 cases. In 5 cases with concomitant aortic valve replacement (AVR), myocardial protection was ensured through antegrade crystalloid cardioplegia with mild hypothermia (32–34 °C). Prior to cardiopulmonary bypass (CPB), heparin was given to achieve an activated clotting time (ACT) of ≥ 400 s. Patients, who underwent UFTA_,_ were rewarmed to a minimal body temperature of >36.5 °C before weaning from CPB. At the end of surgery, all patients were transferred to the ICU.

### Patients groups

Within the mentioned period, the patients were individually selected for the UFTA protocol at the discretion of the attending anesthetists and cardiac surgeons. The exclusion criteria for UFTA included age ≥ 70 years, INTERMACS levels 1 and 2, chronic obstructive pulmonary disease (COPD) > grade II, body mass index ≥ 30 kg/m^2^ (BMI), impaired preoperative pulmonary function with a reduced forced expiratory volume in 1 s (FEV1)/Forced vital capacity (FVC) ratio = FEV1% < 65%, cerebrovascular accident (CVA) in medical history and preoperative hemodialysis. Due to the fact that this is the first systematic approach to this new technique, we deliberately chose a pilot design: Only patients deemed suitable for this new strategy on an expert consensus between surgeon and anesthetist were recruited. This of course poses a source of bias, yet from an ethical point of view, it remains the only plausible strategy to determine non-inferiority before entering a randomized controlled trial design.

**Fig. 1:**
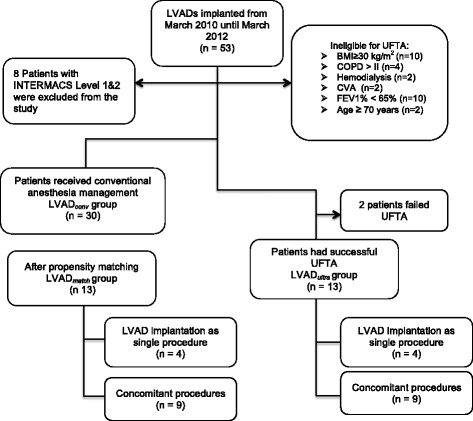
Patients groups and study design. BMI: Body mass index kg/m^2^; COPD: Chronic obstructive lung disease; CVA: Cerebrovascular accident; FEV1%: Ratio of forced expiratory volume in 1 s (FEV1)/ Forced vital capacity (FVC); INTERMACS: Interagency Registry for Mechanically Assisted Circulatory Support; LVAD: Left ventricular assist device; LVAD_*conv*_: All LVAD patients, who received conventional anesthesia; LVAD_*match*_: LVAD patients, who received conventional anesthesia and were matched with the 13 patients who received ultra-fast-track anesthesia; LVAD_*ultra*_: Patients, who had ultra-fast-track anesthesia

To avoid inappropriate comparison, patients classified as INTERMACS Level 1 or 2, were excluded, due to the fact that patients with INTERMACS level 1 and 2 are high risk patients, hemodynamically unstable, some of them already are intubated and on positive inotropic support preoperatively, all other LVAD patients who were extubated according to our regular institutional protocol during the first 12 h. postoperatively or later in the ICU formed our historical control group (LVAD_*conv*_). Patients, who had successful UFTA, formed (LVAD_*ultra*_) group and were retrospectively compared to matched patients from the LVADconv group (LVAD_*match*_) (Fig. 1), for detailed information of patient's data please refer to the Additional file 1. This excludes the two patients who failed UFTA despite an intention to treat. Those cases are further described in the following paragraph *UFTA failure*.

### Anesthesia protocol

All patients scheduled for LVAD implantation received no premedication prior to surgery. Cardiac medication was continued until the morning of surgery. In both groups, anesthesia was induced with sufentanil 0.25–0.5 μg/kg, propofol 1 to 1.5 mg/kg and rocuronium 1 mg/kg. Muscle relaxants were not repeated during the operation. Anesthesia was maintained with propofol 2–4 mg/kg/h and sufentanil 0.5–2.0 μg/kg/h. When the surgeon started the actual LVAD implantation procedure, sufentanil was stopped, and remifentanyl (continuous infusion 0.2 μg/kg/min) was used for analgesia in the LVAD_*ultra*_ group. At the end of surgery, before skin closure, remifentanyl application was stopped, and the patients received piritramid 0.1 mg/kg. Propofol was discontinued, and patients were put in a beach-chair position. A remaining neuromuscular block was excluded. On arousal, the patients were asked to obey simple commands and tasks, e.g., move arms and legs, swallow and lift head. Finally, after negotiation of pain, the patient’s trachea was extubated. Oxygen was given via a facemask (target SpO_2_ 94–100%), and carbon dioxide retention was excluded.

In the conventional group, both sufentanil and propofol were continued in the ICU. These patients were ventilated and weaned from ventilation according to clinical standards, including lung protective ventilation. Extubation criteria included: 1. Normothermia and normovolemia; 2. Absence of surgical bleeding with adequate hemostasis with normal activated coagulation time; 3. Complete reversal of the neuromuscular blockade assessed by limb movements and spontaneous ventilation sufficient to maintain arterial oxygen saturation over 95% with 40% FiO_2_ and end-tidal carbon dioxide under 50 mmHg; 4. Hemodynamic stability without significant inotropic support; and 5. A conscious patient obeying simple verbal commands.

### Hemodynamic monitoring in the OR and ICU

In addition to basic monitoring (ECG, pulse oximetry, invasive blood pressure measurements, temperature measurements and arterial and central venous blood gas analysis with a sampling frequency of 30 min or as determined by clinical protocol), all patients received an additional pulmonary artery catheter (PAC) to control cardiac index (CI) and central venous oxygen saturation (ScvO_2_). Transesophageal echocardiography (TEE) was routinely used in all procedures.

### Definition of RVF

With no universally accepted definition of RVF after LVAD placement, we used the following definition: 1. ≥ 48 h. nitric oxide (NO) (or other pulmonary vasodilator, such as iloprost); 2. Multi-organ failure from persistent hypotension without evidence of sepsis; 3. Positive inotropic agents for ≥14 days post-LVAD or late re-institution of inotropes (>14 days post-LVAD); or 4. Needing right ventricular assist device. This model was used by Kalogeropoulos et al. [19] and is consistent with the Kormos et al. model [14].

### Diagnostic criteria of postoperative delirium

The definition of delirium is based on the 5th edition of the Diagnostic and Statistical Manual of Mental Disorders (DSM-5) from the American Psychiatric Association [20]. We use the Confusion Assessment Method (CAM) for the ICU (CAM-ICU) [21, 22]. The CAM-ICU was estimated for each patient in the ICU at least twice a day during both day and night shift rounds.

### Statistical analyses

Continuous variables are expressed as the means ± standard deviation (SD) and categorical variables as absolute numbers and percentages. Due to non-normally distributed data the comparisons between groups before matching were performed with the Mann-Whitney-U-test for continuous variables and Fisher’s exact test or χ2 test, where appropriate, for categorical variables. Due to the small group of patients who had successful UFTA (LVAD_*ultra*_) and to reduce selection bias, we performed propensity score matching to match all 13 patients in the LVAD_*ultra*_ group with the appropriate patients in the LVAD_*conv*_ group after excluding patients classified as INTERMACS level 1–2 from LVAD_*conv*_ group. Propensity scores were calculated for each patient using multivariate logistic regression based on the following preoperative covariates: Age, BMI, COPD ≤ grade II, FEV1%, peripheral arterial disease (PAD), preoperative creatinine, Re-do procedures, European System for Cardiac Operative Risk Evaluation II (EuroSCORE II), left ventricular ejection fraction (EF), pulmonary artery mean pressure (PAMP), pulmonary capillary wedge pressure (PCWP), CI, and right ventricular end-diastolic basal-diameter from a four chamber view (RVEDD1), measured according to the American Society of Echocardiography guidelines [23]. Variables were chosen for the propensity matching according to known preoperative risk-factors, which promote prolonged mechanical ventilation, prolonged ICU stay after open heart cardiac surgery [10, 24] and right heart failure after LVAD implantation [14, 18, 19]. LVAD_*ultra*_ patients were matched to LVAD_*conv*_ patients with the closest propensity score with the nearest-neighbor algorithm without replacement and with a 0.2 matching tolerance. The LVAD_*conv*_ patients who could be matched formed the matched group LVAD_*match*_. Figure 1 describes the design of the study and the patient groups. Kaplan–Meier analyses were used to estimate the survival functions for patients in both groups. Differences in survival were evaluated using the log-rank test. Patients were censored for transplantation. After matching, categorical outcomes were compared with the McNemar’s test, and continuous outcomes were compared with Wilcoxon signed-rank test. For the comparisons of continuous variables with repeated measurements (CI, ScvO_2_, CVP, MPAP) a One-Way ANOVA test with Sidak’s correction were performed. All statistical analyses were performed using SPSS software, version 23.0 (Chicago, IL, USA). Propensity matching was performed with the extension package of the statistical program R version 3.1. A two-tailed *p*-value of < 0.05 was considered significant. All p-values were reported as three digit numbers.

## Results

A total of 53 patients (16.9% female, mean age 62 ± 7.9) received LVAD implantation (50 Heart Mate II; HMII, Thoratec, Pleasanton, CA, USA and 3 HeartWare HVAD, HeartWare Inc., Framingham, MA, USA). 8 patients, who were categorized in INTERMACS level 1 or 2, were excluded from the study (Fig. 1). 15 patients were eligible for UFTA. UFTA was successfully performed in 13 patients and failed in 2 patients. The two patients, who were not able to be extubated within the first 4 h postoperatively, required high doses of inotropic support at the end of surgery and were hemodynamically unstable, possibly due to systemic inflammatory response syndrome. Demographics and preoperative data are listed in Table 1. Combined surgery was performed in 29 patients; details of procedures and intraoperative data are described in Table 2. Six patients in LVAD_*ultra*_ group and 10 patients in the LVAD_*match*_ group had LVAD implantation as destination therapy (DT). No differences in preoperative risk factors and demographics were detected between LVAD_*ultra*_ and LVAD_*match*_ groups (Table 1). The FEV1% was significantly lower in the LVAD_*conv*_ group compared to the LVAD_*ultra*_ group (LVAD_*conv*_ vs. LVAD_*ultra*_: 64.8 ± 7.1 vs. 74.4 ± 8.5, *p* = 0.001). All patients survived surgery. Patient in LVAD_*conv*_ group had significantly higher body mass index compared to the LVAD_*ultra*_ group (29.1 ± 4.2 Kg/m^2^ vs. 26.1 ± 3.1 Kg/m^2^, *p* = 0.009) and higher preoperative creatinine values (1.3 ± 0.2 mg/dL vs. 1.1 ± 0.3 mg/dL, *p* = 0.032, respectively).

**Table 1:** Demographic and preoperative data

	Unmatched			Matched			
	LVAD_*conv*_	LVAD_*ultra*_	*p-*values	LVAD_*match*_	LVAD_*ultra*_	*p*-values	Patients failed UFTA (*n* = 2)
	*n* = 30	*n* = 13		*n* = 13	*n* = 13		
Age	65.2 ± 8.4	61.1 ± 7.9	0.108	64.1 ± 4.8	61.1 ± 7.9	0.485	63.5 ± 0.5
Female n (%)	6 (20)	2 (15.4)	1.000	1 (7.8)	2 (15.4)	0.500	1(50)
BMI kg/m^2^	29.5 ± 4.2	26.1 ± 3.1	**0.009**	26.3 ± 3.0	26.1 ± 3.1	0.735	25.8 ± 1.2
PAD n (%)	7 (23.3)	5 (38.5)	0.460	5 (38.5)	5 (38.5)	1.000	1(50)
creatinine (mg/dl)	1.3 ± 0.2	1.1 ± 0.3	**0.032**	1.1 ± 0.2	1.1 ± 0.3	0.907	1.1 ± 0.2
GFR mL/min.	65.6 ± 6.4	66.5 ± 6.5	0.673	65.3 ± 6.8	66.5 ± 6.5	0.693	65.5 ± 0.5
Prior HD	2 (6.7)	0	1.000	0	0	-	0
COPD ≤ II n (%)	8 (26.7)	5 (38.5)	0.485	3 (23.1)	5 (38.5)	0.727	1(50)
COPD > II n (%)	4 (6.7)	0	0.297	0	0	-	0
FEV1 %	64.8 ± 7.1	74.4 ± 8.5	**0.001**	68.5 ± 6.2	74.4 ± 8.5	0.068	69.5 ± 1.5
CVA	2 (6.7)	0	1.000	0	0	-	0
DM n (%)	12 (40)	2 (15.3)	0.163	4 (30.8)	2 (15.3)	0.625	2(100)
Nicotine	20 (66.7)	8 (61.5)	0.742	11 (84.6)	8 (61.5)	0.453	2(100)
DCM n (%)	6 (20)	2 (15.4)	1.000	3 (23.1)	2 (15.4)	1.000	0
ICM n (%)	24 (80)	11 (84.6)	1.000	10 (76.7)	11 (84.6)	1.000	2(100)
CI L/min/m^2^	2.4 ± 0.7	3.0 ± 1.6	0.651	2.3 ± 1	3.0 ± 1.6	0.251	2.9 ± 1
MPAP mmHg	28.9 ± 11.4	30.6 ± 11.6	0.758	34.5 ± 9.1	30.6 ± 11.6	0.198	30.5 ± 0.5
PCWP mmHg	19.3 ± 9.8	21.3 ± 9.4	0.614	23.8 ± 8.1	21.3 ± 9.4	0.330	24.5 ± 1.5
LVEF %	21.4 ± 6.7	17.7 ± 4.1	0.088	19.1 ± 4.7	17.7 ± 4.1	0.715	20 ± 2
RVEDD1 mm	38.9 ± 5.7	37.4 ± 6.1	0.389	37.5 ± 4.9	37.4 ± 6.1	0.984	38.5 ± 0.5
TAPSE mm	15.7 ± 3.1	14.7 ± 5.6	0.212	15.8 ± 4.1	14.7 ± 5.6	0.552	16.5 ± 0.5
DT n (%)	18 (60)	6 (46.2)	0.509	10 (76.9.6)	6 (46.1)	0.453	0
BTC/BTT n (%)	12 (40)	7 (53.8)	0.509	3 (23.1)	7 (53.8)	0.453	2(100)
EuroSCORE II %	14.1 ± 11.6	13.4 ± 8.1	0.820	14.2 ± 7.8	13.4 ± 8.1	0.786	15.5 ± 0.5
INTERMACS Level n (%):							
Level 3	14 (46.7)	4(30.8)	0.502	2 (15.4)	4 (30.8)	0.431	0
Level 4	16 (53.3)	9(69.2)	0.502	11 (84.6)	9 (69.2)	0.431	2(100)

Continuous variables are expressed as means ± standard deviation (SD). Categorical variables are expressed as percentages and absolute numbers. Bold writing indicates significance

*BMI* body-mass-index, *BTC* bridge to candidacy, *BTT* bridge to transplantation, *CI* cardiac index, *CPB time* cardiopulmonary bypass time (min.), *COPD* chronic obstructive pulmonary disease, *CVA* cerebrovascular accident, *DCM* dilated cardiomyopathy, *DM* diabetes mellitus, *DT* destination therapy, *EuroSCORE II* European System for Cardiac Operative Risk Evaluation II, *FEV1%* ratio of forced expiratory volume in 1 s (FEV1)/ Forced vital capacity (FVC), *GFR* glomerular filtration rate (mL/min.), *HD* hemodialysis, *ICM* ischemic cardiomyopathy, *INTERMACS* Interagency Registry for Mechanically Assisted Circulatory Support, *LVEF* left ventricular ejection fraction, *MPAP* mean pulmonary artery pressure, *PAD* peripheral artery disease, *PCWP* pulmonary capillary wedge pressure (mmHg), *RVEDD1* right ventricular end-diastolic basal diameter (mm), *TAPSE* tricuspid annular plane systolic excursion (mm)

**Table 2:** Surgical procedures and intraoperative data

Procedures	LVAD_*match*_	LVAD_*ultra*_	*p*-values	LVAD_*conv*._	*p-*values
	*n* = 13	*n* = 13		*n* = 30	LVAD_*conv*_ vs. LVAD_*ultra*_
Re-do OP	0	0	-	4 (13.3)	0.297
LVAD alone n (%)	4 (30.7)	4 (30.7)	1.000	10 (33.3)	1.000
LVAD + CABG n (%)	4 (30.8)	4 (30.7)	1.000	8 (26.7)	1.000
LVAD + TVR n (%)	3 (23)	2 (15.3)	0.984	5 (16.7)	1.000
LVAD + CABG + TVR n (%)	2 (15.4)	2 (15.3)	1.000	1 (3.3)	0.518
LVAD + AVR n (%)	0	1 (7.7)	0.988	4 (13.3)	1.000
LVAD + AVR + CABG n (%)	0	0	-	2 (6.7)	1.000
CPB time min.	140.5 ± 34.8	118.6 ± 29.3	0.381	156.7 ± 56.1	**0.032**
PRBC	2.5 ± 2.5	2.4 ± 2.9	0.945	3.1 ± 2.9	0.459

Bold writing indicates significance

*CABG* coronary artery bypass graft, *LVAD* left ventricular assist device, *TVR* tricuspid valve repair, *AVR* aortic valve replacement, *CPB* cardiopulmonary bypass, *PRBC* packed red blood cells

### Time to extubation and intensive care unit stay

**Fig. 2:**
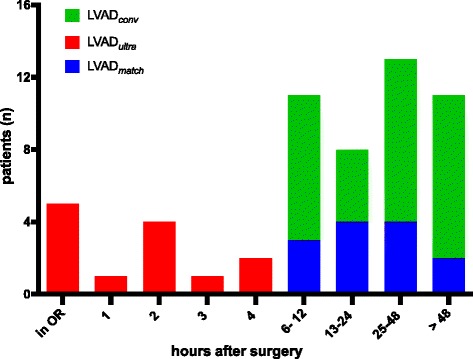
Cumulative number of patients extubated by postoperative hour. OR: Operating room

The mean time to extubation differed significantly between the LVAD_*ultra*_ and LVAD_*match*_ groups (1.2 ± 1.3 h. vs. 42.3 ± 32.1 h., respectively, *p* = 0.002). Five LVAD_*ultra*_ patients (38.5%) were immediately extubated in the OR (Fig. 2). Three LVAD_*match*_ patients were re-intubated due to respiratory failure compared with one LVAD_*ultra*_ patient (*p* = 0.125). Eight patients in LVAD_*conv*_ group required re-intubation (*p* = 0.236).

LVAD_*ultra*_ patients had significantly shorter ICU stays than LVAD_*match*_ patients (LVAD_*ultra*_: 60.2 ± 43.4 h. vs. LVAD_*match*_: 153.1 ± 95.9 h., *p* = 0.016) and required significantly shorter periods of inotropic support (LVAD_*ultra*_: 15.9 ± 19.5 h. vs. LVAD_*match*_: 88.5 ± 108 h., *p* = 0.001). There was a tendency for shorter hospital length (LOS) of stay for the LVAD_*ultra*_ patients (LVAD_*ultra*_: 22.1 ± 9.5 days vs. LVAD_*match*_: 26.3 ± 14.9 days, *p* = 0.055). The LOS of LVAD_*ultra*_ patients was significantly shorter compared with the LVAD_*conv*_ (22.1 ± 9.5 days vs. 37.8 ± 23.6, *p* = 0.026).

### Postoperative complications

Postoperative data are described in Table 3. LVAD_*ultra*_ patients had lower incidence of pneumonia (7.7% vs. 46.5%, *p* = 0.031) compared to LVAD_*match*_ patients. There was also a tendency for lower incidence of postoperative sepsis in the LVAD_*ultra*_ group compared with LVAD_*match*_ group (0 vs. 23.1%. p = 0.250). None of the LVAD_*ultra*_ patients developed postoperative delirium, while six patients in LVAD_*match*_ group developed postoperative delirium (*p* = 0.031). The glomerular filtration rate (GFR) measured 24 h. postoperatively was higher in the LVAD_*ultra*_ group but did not differ significantly compared to the matched group (LVAD_*ultra*_ vs. LVAD_*match*_: 62.8 ± 10.2 vs. 58.1 ± 11.1 mL/min., *p* = 0.331).

**Table 3:** Postoperative data

	LVAD_*match*_	LVAD_*ultra*_	*p*-values	LVAD_*conv*_	*p-*values
	*n* = 13	*n* = 13	LVAD_*match*_ vs. LVAD_*ultra*_	*n* = 30	LVAD_*conv*_ vs. LVAD_*ultra*_
Time to extubation in hr.	42.3 ± 32.1	1.2 ± 1.3	**0.0002**	56.5 ± 67.7	**0.0001**
Re-intubation n (%)	3 (23.1)	1(7.7)	0.125	8(26.7)	0.236
ICU stays in hrs n (%)	153.1 ± 95.9	60.2 ± 43.3	**0.016**	186.1 ± 163.1	**0.0008**
Hospital LOS in days after Implantation	26.3 ± 14.9	22.1 ± 9.5	0.055	37.8 ± 23.6	**0.026**
Pneumonia n (%)	7 (46.5)	1 (7.7)	**0.031**	10 (33.3)	0.129
Sepsis n (%)	3 (23.1)	0	0.250	7 (23.3)	0.082
Delirium n (%)	6 (46.5)	0	**0.031**	14 (46.7)	**0.003**
Hemodialysis n (%)	3 (23.1)	0	0.250	8 (26.7)	0.081
GFR 24 h. post-op mL/min.	58.1 ± 11.1	62.8 ± 10.2	0.331	51.9 ± 16.5	**0.033**
Re-thoracotomy n (%)	0	0	-	3 (10)	0.541
Inotropic support in hr.	88.5 ± 108	15.9 ± 19.5	**0.001**	113.3 ± 120.2	**0.001**
RVF n (%)	6 (46.5)	0	**0.031**	8 (26.7)	0.081
RV-ECMO n (%)	0	0	-	5 (16.7)	0.300

Bold writing indicates significance

*GFR* glomerular filtration rate (mL/min.), *ICU* intensive care unit, *LOS* length of stay, *Post-op* postoperative, *RV-ECMO* extracorporeal membrane oxygenation as a right ventricular assist device, *RVF* right ventricular failure

Interestingly, none of the LVAD_*ultra*_ patients developed RVF in the first 30 postoperative days (POD), whereas six LVAD_*match*_ patients developed RVF (*p* = 0.031). Of the six patients, who developed RVF in the LVAD_*match*_ group; One required implantation of extracorporeal membrane oxygenation as a temporary right ventricular assist device (RV-ECMO); Two patients required prolonged use of pulmonary vasodilator (NO) > 48 h.; One patient required prolonged use of positive inotropic agents ≥14 postoperative days; Two patients needed ICU re-admission with requirement of late positive inotropic support.

### Hemodynamic parameters in the first 24 h. after surgery

**Fig. 3:**
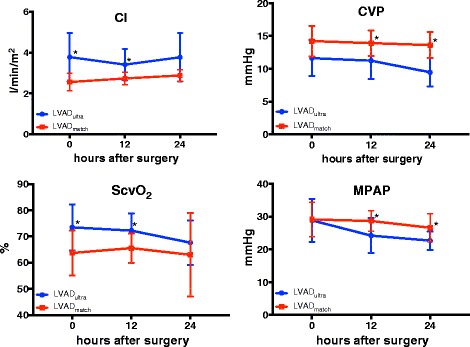
Hemodynamic parameters during 24 h postoperative. *: indicates significance; CI: cardiac index L/min/m^2^; CVP: central venous pressure mmHg, ScvO_2_: central venous saturation %; MPAP: mean pulmonary artery pressure mmHg. *P*-values were carried out with one-way ANOVA test with Sidak’s correction

An overview of the hemodynamic parameters is listed in Table 4 and in Fig. 3. At ICU admission, CI and central venous saturation (ScvO_2_) of the LVAD_*ultra*_ group were significantly higher than those of the LVAD_*match*_ group, (LVAD_*ultra*_: 3.7 ± 1.1 vs. LVAD_*match*_: 2.6 ± 0.4 L/min/m^2^, *p* = 0.013 and LVAD_*ultra*_: 73.4 ± 4.7% vs. LVAD_*match*_: 63.7 ± 8.6%, *p* = 0.028). The difference in CI and ScvO_2_ between the two matched groups was still significant at 12 h. but at 24 h postoperatively no significant difference could be detected in CI and ScvO_2_ between the matched group (LVAD_*ultra*_: 3.4 ± 0.7 vs. LVAD_*match*_: 2.8 ± 0.3 L/min/m^2^, *p* = 0.017 and LVAD_*ultra*_: 72.2 ± 6.5 vs. LVAD_*match*_: 65.5 ± 5.6%, *p* = 0.034). CVP and MPAP did not differ significantly at ICU admission; however, at 12 h. and 24 h. postoperatively, LVAD_*ultra*_ patients had significantly lower CVP and MPAP compared to LVAD_*match*_ patients (Table 4 and Fig. 3).

**Table 4:** Postoperative Hemodynamic parameter

	LVAD_*match*_	LVAD_*ultra*_	*p*-values
Admission CI L/min/m^2^	2.6 ± 0.4	3.7 ± 1.1	**0.013**
12 h CI L/min/m^2^	2.8 ± 0.3	3.4 ± 0.7	**0.017**
24 h CI L/min/m^2^	2.9 ± 0.3	3.5 ± 0.7	0.078
Admission ScvO_2_ %	63.7 ± 8.6	73.4 ± 4.7	**0.028**
12 h ScvO_2_ %	65.5 ± 5.6	72.2 ± 6.5	**0.034**
24 h ScvO_2_ %	62.6 ± 15.4	68.9 ± 4.3	0.759
Admission CVP mmHg	14.2 ± 2.3	11.6 ± 2.7	0.071
12 h CVP mmHg	13.9 ± 1.9	11.2 ± 1.2	**0.049**
24 h CVP mmHg	13.6 ± 1.9	9.4 ± 2.1	**0.005**
Admission MPAP mmHg	29.2 ± 5.2	28.8 ± 6.5	0.996
12 h MPAP mmHg	28.7 ± 3.2	24.2 ± 5.3	**0.048**
24 h MPAP mmHg	26.6 ± 4.3	22.7 ± 2.9	**0.003**

*P*-values were carried out with one-way ANOVA test with Sidak’s correction; Bold writing indicates significance

*CI* cardiac index L/min/m^2^, *CVP* central venous pressure mmHg, *ScvO*_*2*_ central venous saturation %, *MPAP* mean pulmonary artery pressure mmHg

### Survival after surgery

There was no significant difference in the mean survival months after implantation between the LVAD_*ultra*_ and LVAD_*match*_ groups (37.9 ± 20.7 and 49.5 ± 12.8, respectively, *p* = 0.150). The 30-day mortality was 7.7% in the LVAD_*match*_ group (*n* = *1*) vs. 0 in the LVAD_*ultra*_ group. The patient who died during the first 30 POD had RVF and was treated with RV-ECMO, but the clinical situation was then complicated by an additional septic shock and the patient died from multi-organ failure. There was no significant difference in the one-year and three-year survival after implantation between LVAD_*ultra*_ and LVAD_*match*_; 85% survived in each group after 1 year of implantation, while 69% of LVAD_*ultra*_ patients and 64% of LVAD_*match*_ patients survived after 3 years of LVAD implantation.

**Fig. 4:**
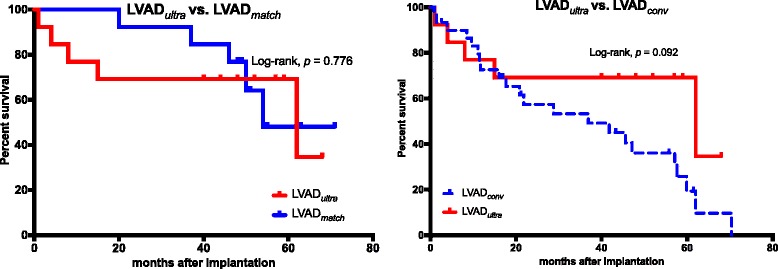
Survival proportions. CI: cardiac index L/min/m^2^; CVP: central venous pressure mmHg, ScvO_2_: central venous saturation %; MPAP: mean pulmonary artery pressure mmHg. Bold writing indicates significance

Kaplan-Meier survival analysis for the follow-up period from March, 2010 until March, 2016 did not reveal any significant difference in survival between the LVAD_*ultra*_ and LVAD_*match*_ groups (log-rank *p* = 0.776, Fig. 4).

The Kaplan-Meier plots between LVAD_*ultra*_ and LVAD_*conv*_ revealed no difference in survival between the LVAD_*conv*_ and LVAD_*ultra*_ groups (log-rank *p* = 0.092), Fig. 4.

## Discussion

This pilot study demonstrate that UFTA after LVAD implantation in INTERMACS level 3–4 patients is feasible and results in a lower incidence of postoperative complications and shorter ICU stay in selected patients. Therefore, our findings agree with other studies examining the feasibility of UFTA in cardiac surgery and heart transplantation albeit preoperative risk factors [5–8].

### Postoperative complications

Previous studies did demonstrate that prolonged mechanical ventilation is associated with worse outcomes and higher mortality [9, 10, 25]. Cheng et al. found in a large randomized trial that prolonged mechanical ventilation results in worse physiologic outcomes as a result of atelectasis and intrapulmonary shunting [26]. In our study the incidences of pneumonia was significantly lower in the LVAD_*ultra*_ group versus the LVAD_*match*_ group. These findings support those of Kurihara et al. and Kradzalic et al. [27, 28], who demonstrated a lower incidence of ventilator-associated pneumonia after FTA. Despite that the incidence of sepsis did not differ significantly between the two matched groups, a tendency for lower incidence of sepsis in the LVAD_*ultra*_ could be detected (*p* = 0.055). Most importantly we could not detect any cases of postoperative delirium in the LVAD_*ultra*_ group, while six patients in the LVAD_*match*_ group had postoperative delirium, which is a risk factor for prolonged ICU stay, especially in cardiac surgery patients. This is consistent with Cheng et al.’s results showing that patients had better results in mini-mental state testing after FTA and returned faster to baseline performance [29]. Previous studies did demonstrate that mechanical ventilation increases the risk of acute kidney failure [30, 31]. Despite the fact that the postoperatively GFR did not differ significantly between the two matched groups, there was a tendency for higher values in the LVAD_*ultra*_ group with none of the patients in the LVAD_*ultra*_ group requiring hemodialysis in the postoperative course.

Patients with end-stage heart failure, who require LVAD implantation, already have a limited tolerance of activity and loss of functional ability preoperatively. These patients had a high risk of morbidity and mortality when developing postoperative complications such as respiratory failure requiring prolonged mechanical support. Our results clearly demonstrate that LVADs patients had lower incidence of postoperative complication after UFTA. UFTA patients could be mobilized and discharged earlier from the ICU. Taken together, these factors have a markedly beneficial impact on the outcome of these severely ill patients accelerating the rehabilitation process [16, 32].

### Incidence of RVF and hemodynamic performance

None of the LVAD_*ultra*_ patients developed RVF, while six LVAD_*match*_ patients did. Early extubation and significantly shorter mechanical ventilation time are considered protective for the right ventricle; prolonged mechanical ventilation is a risk factor for RVF following LVAD implantation [33]. In the nineties, Jardin et al. showed a significant reduction in right ventricular stroke volume (RVS) during mechanical ventilation due to an increase in right ventricular (RV) afterload [34]. Studies of patients with acute respiratory distress syndrome (ARDS) revealed that mechanical ventilation affects RV function due to changes in RV impedance, preload and afterload, significantly affecting mortality [35–37]. RVF after LVAD implantation occurs in 10 to 40% of cases, and RVF results in higher mortality rates [15, 17, 38]. Our results demonstrate that selected patients with end-stage heart failure electively scheduled for LVAD implantation benefit from UFTA due to shorter cardiopulmonary impairment during mechanical ventilation preserving right ventricular function.

Indeed, CI differed significantly between the groups directly after LVAD implantation, but this effect vanished 24 h. after surgery. Most importantly, CVP values were significantly lower in the LVAD_*ultra*_ group at 12 and 24 h. after surgery. These findings contrast with Meissner et al., who did not record any significant hemodynamic differences in either CI or CVP between fast-track-anesthesia (FTA) and conventional anesthesia (CA) following cardiac surgery in children [7]. Similarly, Djaiani et al. found no significant differences in cardiac output between UFTA and CA after adult cardiac surgery [8]. In accordance with our results, Morales et al. found significant improvement of hemodynamic performance after UFTA in children after Fontan’s procedure [39], and Kurihara et al. mentioned significantly lower CVPs after FTA compared to CA following cardiac surgery in children [28]. Our results clearly show that UFTA improves hemodynamics and reduces CVP after LVAD implantation in selected patients. This may help preserve RV function as many previous studies found that CVP ≥ 14 mmHg is a risk factor for RVF [14, 18, 19, 40].

### UFTA failure

Previous studies revealed that reduced renal function, hypertension, age, EuroSCORE, cardiopulmonary bypass time, and cross-clamp time are risk factors predicting failure of fast track anesthesia [41, 42]. In our study the two patients, who failed to be extubated within the first 4 h. postoperatively were 64 and 63 years old, both of them had concomitant procedures (LVAD+ TKR and LVAD+ CABG) but the CPB time did not differ significantly compared to the LVAD_*ultra*_ patients (127.6 ± 3.1 vs. 118.6 ± 29.3, *p* = 0.680). Also EuroSCORE II was not significantly higher compared to the LVAD_*ultra*_ group, FEV1% was 68% and 71% in the same range of the LVAD_*ultra*_ patients and GFR did not differ between the two patients and the rest of LVAD_*ultra*_ patients (Table 1). Due to hemodynamic instability and requirement of high doses inotropic support, possibly due to systemic inflammatory response syndrome, the two patients were not able to be extubated within 4 h. postoperatively. These 2 patients were extubated 16 and 22 h. postoperatively. Due to the small group of patients, who failed UFTA in our study, multivariate regression analyzes could not be performed to detect risk factors predicting the failure of UFTA.

### Limitations of the study

Deliberate selection of patients remains a strong source of bias that cannot be controlled by propensity matching. Careful selection of patients will remain necessary for UFTA procedure and future studies will have to determine coherent selection criteria. The retrospective nature of our study prevented standardization between the two groups. Accordingly, multivariate regression analyses to determine risk factors contributing to the failure of UFTA could not be performed. Adequate patient selection is vital when implementing fast-track regimens in perioperative LVAD protocols. The small number of matched pairs between both groups may limit generalization of the results. However, given the results of our study, prospective trials are encouraged to support broader application of UFTA in LVAD therapy.

## Conclusion

In this pilot study, we demonstrated the feasibility of ultra-fast-track anesthesia in LVAD implantation in patients with INTERMACS level 3–4. Patients had a lower incidence of postoperative complications, better hemodynamic performance, shorter length of ICU stay and lower incidence of RVF after UFTA. Prospective investigations are encouraged to evaluate the capability of UFTA for sustainable protection of right ventricular function, and these studies should aim to identify useful criteria for adequate patient stratification.

## Additional file

Additional file 1: Detailed information of patient's data. (XLSX 56 kb)

## Abbreviations

ACT: Activated clotting time
AVR: Aortic valve replacement
BMI: Body mass index Kg/m^2^
CABG: Coronary artery bypass graft
CI: Cardiac index L/min/m^2^
COPD: Chronic obstructive pulmonary disease
CPB: Cardiopulmonary bypass
CVA: Cerebrovascular accident
CVP: Central venous pressure
ECG: Electro cardiogram
ECMO: Extracorporeal membrane oxygenation
EF: Ejection fraction %
EuroSCORE II: European System for Cardiac Operative Risk Evaluation II
FEV1: Forced expiratory volume in 1 s
FTA: Fast-track anesthesia
FVC: Forced vital capacity
GFR: Glomerular filtration rate
ICU: Intensive care unit
INTERMACS: Interagency Registry for Mechanically Assisted Circulatory Support
LVAD: Left ventricular assist device
NO: Nitric oxide
OR: Operating room
PAC: Pulmonary artery catheter
PAD: Peripheral arterial disease
PAMP: Pulmonary artery mean pressure mmHg
PCWP: Pulmonary capillary wedge pressure mmHg
POD: Postoperative day
PRBCs: Packed red blood cells
RVEDD1: Right ventricular end-diastolic basal-diameter mm
RVF: Right ventricular failure
ScvO_2_: Central venous saturation
TEE: Transesophageal echocardiography
TVR: Tricuspid valve repair
UFTA: Ultra-fast-track anesthesia

## References

[1] Zhu F, Lee A, Chee YE (2012). Fast-track cardiac care for adult cardiac surgical patients. Cochrane Database Syst Rev.

[2] Svircevic V, Nierich AP, Moons KG, Brandon Bravo Bruinsma GJ, Kalkman CJ, van Dijk D (2009). Fast-track anesthesia and cardiac surgery: a retrospective cohort study of 7989 patients. Anesth Analg.

[3] Cheng DCH, Wall C, Djaiani G, Peragallo RA, Carroll J, Li C (2003). Randomized assessment of resource use in fast-track cardiac surgery 1-year after hospital discharge. Anesthesiology.

[4] Plumer H, Markewitz A, Marohl K, Bernutz C, Weinhold C (1998). Early extubation after cardiac surgery: a prospective clinical trial including patients at risk. Thorac Cardiovasc Surg.

[5] Kianfar AA, Ahmadi ZH, Mirhossein SM, Jamaati H, Kashani BS, Mohajerani SA (2015). Ultra fast-track extubation in heart transplant surgery patients. Int J Crit Illn Inj Sci.

[6] Borracci RA, Ochoa G, Ingino CA, Lebus JM, Grimaldi SV, Gambetta MX. Routine operation theatre extubation after cardiac surgery in the elderly. Interact Cardiovasc Thorac Surg. 2016. doi:10.1093/icvts/ivv409. 10.1093/icvts/ivv409 PMC4892145 26826715

[7] Meissner U, Scharf J, Dotsch J, Schroth M (2008). Very early extubation after open-heart surgery in children does not influence cardiac function. Pediatr Cardiol.

[8] Djaiani GN, Ali M, Heinrich L, Bruce J, Carroll J, Karski J (2001). Ultra-fast-track anesthetic technique facilitates operating room extubation in patients undergoing off-pump coronary revascularization surgery. J Cardiothorac Vasc Anesth.

[9] Hillis LD, Smith PK, Anderson JL, Bittl JA, Bridges CR, Byrne JG (2011). 2011 ACCF/AHA Guideline for Coronary Artery Bypass Graft Surgery: a report of the American College of Cardiology Foundation/American Heart Association Task Force on Practice Guidelines. Circulation.

[10] Reddy SL, Grayson AD, Griffiths EM, Pullan DM, Rashid A (2007). Logistic risk model for prolonged ventilation after adult cardiac surgery. Ann Thorac Surg.

[11] Cherpanath TG, Lagrand WK, Schultz MJ, Groeneveld AB (2013). Cardiopulmonary interactions during mechanical ventilation in critically ill patients. Neth Heart J.

[12] Cournand A, Motley HL (1948). Physiological studies of the effects of intermittent positive pressure breathing on cardiac output in man. Am J Physiol.

[13] Luecke T, Pelosi P (2005). Clinical review: positive end-expiratory pressure and cardiac output. Crit Care.

[14] Kormos RL, Teuteberg JJ, Pagani FD, Russell SD, John R, Miller LW (2010). Right ventricular failure in patients with the HeartMate II continuous-flow left ventricular assist device: incidence, risk factors, and effect on outcomes. J Thorac Cardiovasc Surg.

[15] MacGowan GA, Schueler S (2012). Right heart failure after left ventricular assist device implantation: early and late. Curr Opin Cardiol.

[16] Yuan N, Arnaoutakis GJ, George TJ, Allen JG, Ju DG, Schaffer JM (2012). The spectrum of complications following left ventricular assist device placement. J Card Surg.

[17] Argiriou M, Kolokotron SM, Sakellaridis T, Argiriou O, Charitos C, Zarogoulidis P (2014). Right heart failure post left ventricular assist device implantation. J Thorac Dis.

[18] Lampert BC, Teuteberg JJ. Right ventricular failure after left ventricular assist devices. J Heart Lung Transplant.34(9):1123–30. doi:10.1016/j.healun.2015.06.015. 10.1016/j.healun.2015.06.015 26267741

[19] Kalogeropoulos AP, Kelkar A, Weinberger JF, Morris AA, Georgiopoulou VV, Markham DW (2015). Validation of clinical scores for right ventricular failure prediction after implantation of continuous-flow left ventricular assist devices. J Heart Lung Transplant.

[20] American Psychiatric Association (2013). American Psychiatric Association. DSM-5 Task Force. Diagnostic and statistical manual of mental disorders : DSM-5.

[21] Ely EW, Margolin R, Francis J, May L, Truman B, Dittus R (2001). Evaluation of delirium in critically ill patients: validation of the Confusion Assessment Method for the Intensive Care Unit (CAM-ICU). Crit Care Med.

[22] Inouye SK, van Dyck CH, Alessi CA, Balkin S, Siegal AP, Horwitz RI (1990). Clarifying confusion: the confusion assessment method. A new method for detection of delirium. Ann Intern Med.

[23] Rudski LG, Lai WW, Afilalo J, Hua L, Handschumacher MD, Chandrasekaran K (2010). Guidelines for the echocardiographic assessment of the right heart in adults: a report from the American Society of Echocardiography endorsed by the European Association of Echocardiography, a registered branch of the European Society of Cardiology, and the Canadian Society of Echocardiography. J Am Soc Echocardiogr.

[24] Ettema RG, Peelen LM, Schuurmans MJ, Nierich AP, Kalkman CJ, Moons KG (2010). Prediction models for prolonged intensive care unit stay after cardiac surgery: systematic review and validation study. Circulation.

[25] Rashid A, Sattar KA, Dar MI, Khan AB (2008). Analyzing the outcome of early versus prolonged extubation following cardiac surgery. Ann Thorac Cardiovasc Surg.

[26] Cheng DC, Karski J, Peniston C, Asokumar B, Raveendran G, Carroll J (1996). Morbidity outcome in early versus conventional tracheal extubation after coronary artery bypass grafting: a prospective randomized controlled trial. J Thorac Cardiovasc Surg.

[27] Krdzalic A, Kosjerina A, Jahic E, Rifatbegovic Z, Krdzalic G (2013). Influence of Remifentanil/Propofol Anesthesia on Ventilator-associated Pneumonia Occurence After Major Cardiac Surgery. Med Arch.

[28] Kurihara Y, Shime N, Miyazaki T, Hashimoto S, Tanaka Y (2009). Clinical and hemodynamic factors associated with the outcome of early extubation attempts after right heart bypass surgery. Interact Cardiovasc Thorac Surg.

[29] Cheng DC (1998). Fast-track cardiac surgery: economic implications in postoperative care. J Cardiothorac Vasc Anesth.

[30] van den Akker JP, Egal M, Groeneveld AB (2013). Invasive mechanical ventilation as a risk factor for acute kidney injury in the critically ill: a systematic review and meta-analysis. Crit Care.

[31] Hering R, Peters D, Zinserling J, Wrigge H, von Spiegel T, Putensen C (2002). Effects of spontaneous breathing during airway pressure release ventilation on renal perfusion and function in patients with acute lung injury. Intensive Care Med.

[32] Perme CS, Southard RE, Joyce DL, Noon GP, Loebe M (2006). Early mobilization of LVAD recipients who require prolonged mechanical ventilation. Tex Heart Inst J.

[33] Ochiai Y, McCarthy PM, Smedira NG, Banbury MK, Navia JL, Feng J (2002). Predictors of severe right ventricular failure after implantable left ventricular assist device insertion: analysis of 245 patients. Circulation.

[34] Jardin F, Delorme G, Hardy A, Auvert B, Beauchet A, Bourdarias JP (1990). Reevaluation of hemodynamic consequences of positive pressure ventilation: emphasis on cyclic right ventricular afterloading by mechanical lung inflation. Anesthesiology.

[35] Bouferrache K, Vieillard-Baron A (2011). Acute respiratory distress syndrome, mechanical ventilation, and right ventricular function. Curr Opin Crit Care.

[36] Vieillard-Baron A, Loubieres Y, Schmitt JM, Page B, Dubourg O, Jardin F (1999). Cyclic changes in right ventricular output impedance during mechanical ventilation. J Appl Physiol (1985).

[37] Jardin F, Vieillard-Baron A (2007). Is there a safe plateau pressure in ARDS? The right heart only knows. Intensive Care Med.

[38] Dang NC, Topkara VK, Mercando M, Kay J, Kruger KH, Aboodi MS et al. Right Heart Failure After Left Ventricular Assist Device Implantation in Patients With Chronic Congestive Heart Failure. J Heart Lung Transplant. 25(1):1–6. doi:10.1016/j.healun.2005.07.008. 10.1016/j.healun.2005.07.008 16399523

[39] Morales DLS, Carberry KE, Heinle JS, McKenzie ED, Fraser CD, Jr., Diaz LK. Extubation in the Operating Room After Fontan's Procedure: Effect on Practice and Outcomes. Ann Thorac Surg. 86(2):576–82. doi:10.1016/j.athoracsur.2008.02.010. 10.1016/j.athoracsur.2008.02.010 18640336

[40] Atluri P, Goldstone AB, Fairman AS, MacArthur JW, Shudo Y, Cohen JE (2013). Predicting right ventricular failure in the modern, continuous flow left ventricular assist device era. Ann Thorac Surg.

[41] Youssefi P, Timbrell D, Valencia O, Gregory P, Vlachou C, Jahangiri M (2015). Predictors of failure in fast-track cardiac surgery. J Cardiothorac Vasc Anesth.

[42] Widyastuti Y, Stenseth R, Pleym H, Wahba A, Videm V (2012). Pre-operative and intraoperative determinants for prolonged ventilation following adult cardiac surgery. Acta Anaesthesiol Scand.

